# Multimodal Discrimination of Schizophrenia Using Hybrid Weighted Feature Concatenation of Brain Functional Connectivity and Anatomical Features with an Extreme Learning Machine

**DOI:** 10.3389/fninf.2017.00059

**Published:** 2017-09-08

**Authors:** Muhammad Naveed Iqbal Qureshi, Jooyoung Oh, Dongrae Cho, Hang Joon Jo, Boreom Lee

**Affiliations:** ^1^Department of Biomedical Science and Engineering, Institute of Integrated Technology, Gwangju Institute of Science and Technology Gwangju, South Korea; ^2^Department of Neurologic Surgery, Mayo Clinic Rochester, MN, United States

**Keywords:** Schizophrenia, COBRE, neuroimaging, global functional connectivity, group ICA, hybrid weighted feature concatenation, machine learning

## Abstract

Multimodal features of structural and functional magnetic resonance imaging (MRI) of the human brain can assist in the diagnosis of schizophrenia. We performed a classification study on age, sex, and handedness-matched subjects. The dataset we used is publicly available from the Center for Biomedical Research Excellence (COBRE) and it consists of two groups: patients with schizophrenia and healthy controls. We performed an independent component analysis and calculated global averaged functional connectivity-based features from the resting-state functional MRI data for all the cortical and subcortical anatomical parcellation. Cortical thickness along with standard deviation, surface area, volume, curvature, white matter volume, and intensity measures from the cortical parcellation, as well as volume and intensity from sub-cortical parcellation and overall volume of cortex features were extracted from the structural MRI data. A novel hybrid weighted feature concatenation method was used to acquire maximal 99.29% (*P* < 0.0001) accuracy which preserves high discriminatory power through the weight of the individual feature type. The classification was performed by an extreme learning machine, and its efficiency was compared to linear and non-linear (radial basis function) support vector machines, linear discriminant analysis, and random forest bagged tree ensemble algorithms. This article reports the predictive accuracy of both unimodal and multimodal features after 10-by-10-fold nested cross-validation. A permutation test followed the classification experiment to assess the statistical significance of the classification results. It was concluded that, from a clinical perspective, this feature concatenation approach may assist the clinicians in schizophrenia diagnosis.

## Introduction

Schizophrenia is a major psychiatric disorder that reportedly affects one percent of the population (Mao et al., 2009). This disorder can cause chronic impairments in cognition, perception, reality testing, and emotion among others (Bhugra, 2005). Its underlying mechanism is still unclear, even though it is considered to involve structural and functional brain abnormalities (Ho et al., 2003; Karlsgodt et al., 2010; Oh et al., 2015, 2017). Schizophrenia is usually diagnosed on the basis of history and mental status examination by psychiatrists under the guidelines of specified diagnostic criteria. Given that the diagnostic process is mainly performed by assessment of visible symptoms and the criteria require long-term observation to evaluate, a standard diagnostic test is needed to reduce the possibility of misdiagnosis.

Nowadays, there is growing interest in the use of machine learning techniques for the diagnosis of diseases (Mirzaei et al., 2016; Qureshi et al., 2016). Especially psychiatric disorders, including schizophrenia, have been the focus of research on automatic diagnosis by machine learning techniques and neuroimaging data (Davatzikos et al., 2005; Fan et al., 2008; Nieuwenhuis et al., 2012; Schnack et al., 2014). Previous studies have mainly utilized unimodal data acquired by structural magnetic resonance imaging (MRI), resting state functional MRI, task related functional MRI, or diffusion MRI, although some studies have included multimodal neuroimaging data. Considering that patients with schizophrenia have both structural and functional abnormalities, multimodal image data can provide more information, enhancing the accuracy of diagnosis. To wit, multimodal data can provide information for classification that is unavailable when using unimodal data. However, it is still unclear whether multimodal data can boost accuracy effectively as previous multimodal classification studies included relatively small samples with less than 35 participants in each group (Du et al., 2012; Ota et al., 2013; Sui et al., 2013).

In this study, we classified patients with schizophrenia and healthy controls using both anatomical (sMRI) and resting state functional (rs-fMRI) features. To date, few studies have used combined sMRI and rs-fMRI features to discriminate patients with schizophrenia from healthy controls (Silva et al., 2014). In addition, previous studies combining sMRI and fMRI utilized only a few parameters such as independent component analysis (ICA) features and gray matter densities. Considering the accuracy was under 95% in these studies, the classifier may be ameliorated with use of more parameters such as global connectivity, surface area, and curvature features among others. Although there exist many previous studies using ICA for extracting the features for classification, however, to the best of our knowledge, the group ICA method used in the present study has never been used before for this purpose.

To utilize multiple modalities, they should be integrated elaborately and the calculation of the importance of each feature type is crucial for high accuracy acquisition. Concurrent with this, we applied and proposed a simple and hybrid weighted feature concatenation method. The method is simple and robust, and can automatically calculate the weight of each feature type. In addition, we compared the accuracy of five types of classifier [linear and non-linear (radial basis function) support vector machine (SVM), linear extreme learning machine (ELM), linear discriminant analysis (LDA), and random forest bagged tree classifier] by applying multimodal brain features and the proposed feature concatenation method. We hypothesized that the combination of whole brain sMRI and rs-fMRI features would be superior to the utilization of unimodal data, and the accuracy would be sufficient for use in clinical practice.

The remainder of this paper is organized as follows: the materials and methods section provides information on the dataset, subject selection, preprocessing of the s/fMRI data, feature extraction, structural measures, global connectivity, group ICA, the hybrid weighted feature concatenation method, the introduction of the classification algorithms, and permutation testing for assessing the significance of the results. The results section presents the comparative results of both the unimodal and multimodal binary classifiers using the ELM, SVM-L, SVM-RBF, LDA, and Random forest classifiers. The discussion section includes the commentary on the results, comparison with previous research, and the clinical significance of the results. The conclusion section includes the limitations of the present study and concludes the paper.

## Materials and methods

### Subjects

We used 72 subjects each from the normal control and schizophrenia group from the Center for Biomedical Research Excellence (COBRE) dataset (Calhoun et al., 2011; Hanlon et al., 2011; Mayer et al., 2013; Stephen et al., 2013). Age ranged from 18 to 65 years in each group. Information on this dataset is available at http://cobre.mrn.org/. A structured clinical interview based on DSM-IV was used by trained clinical psychiatrists for the diagnosis of patients with Schizophrenia in this dataset. For making a balanced study design, we used only 72 subjects from each subgroup of the COBRE dataset.

All the subjects were screened and excluded if they had a history of substance abuse or dependence present in the 12 months prior to the scanning date. In addition, the patients were also screened for history of neurological disorders, intellectually disabled, and severe head trauma with a loss of consciousness for more than 5 min. COBRE is a publically available dataset distributed with “Creative Commons License: Attribution—Non-Commercial” and written informed consent was obtained from all subjects in accordance with the institutional review board (IRB) protocols of the University of New Mexico (UNM) (Cabral et al., 2016). Table 1 shows the demographic information of the subjects.

**Table 1:** Demographic information of subjects from COBRE dataset.

**Groups**	**Normal control**	**Schizophrenia**
No of Subjects	72	72
Age (mean ± STD)	35.875 ± 11.74	38.167 ± 13.894
Sex (Male/Female)	58/14	52/20
Handedness (Left/Right)	10/62	3/69
Age of onsets (years)	–	21.17 ± 7.51
Illness duration (years)	–	9.03 ± 9.88
PANSS positive	–	14.96 ± 4.83
PANSS negative	–	14.53 ± 4.83
PANSS general	–	29.22 ± 8.34
PANSS total	–	58.71 ± 13.75

*STD, Standard deviation; PANSS, positive and negative syndrome scale*.

### Dataset and preprocessing

#### Structural data acquisition

All the participants were scanned with a Siemens TIM 3.0-Tesla scanner. A multi-echo MPRAGE (MEMPR) sequence was used with the following parameters: TR/TE/TI = 2530/[1.64, 3.5, 5.36, 7.22, 9.08]/900 ms, flip angle = 7°, FOV = 256 × 256 mm, Slab thickness = 176 mm, Matrix = 256 × 256 × 176, Voxel size = 1 × 1 × 1 mm, Number of echoes = 5, Pixel bandwidth = 650 Hz, Total scan time = 6 min. Using 5 echoes, the TR, TI, and time to encode partitions for the MEMPR are similar to that of a conventional MPRAGE, resulting in similar GM/WM/CSF contrast.

#### Structural data preprocessing

Cortical reconstruction and volumetric segmentation were performed with the FreeSurfer v. 5.3.0 image analysis suite, which is documented and freely available for download online at http://surfer.nmr.mgh.harvard.edu/. The technical details of these procedures have been described previously (Dale and Sereno, 1993; Dale, 1999; Fischl et al., 1999a,b, 2001, 2002, 2004; Fischl and Dale, 2000; Ségonne et al., 2004; Han et al., 2006; Reuter et al., 2012). Briefly, the preprocessing procedure includes motion correction of volumetric T1-weighted images, removal of non-brain tissue using a hybrid watershed/surface deformation procedure (Ségonne et al., 2004), and automated Talairach transformation. Moreover, the procedure includes the segmentation of subcortical white matter and deep gray matter volumetric structures including the hippocampus, amygdala, caudate nucleus, putamen, ventricles (Fischl et al., 2002, 2004); intensity normalization, tessellation of the gray-white matter boundary, and automated topology correction (Fischl et al., 2001; Ségonne et al., 2007). In addition, surface deformation following intensity gradients was performed to optimally place the gray/white and gray/cerebrospinal fluid (CSF) borders at the location where the greatest shift in intensity defines the transition to the other tissue class (Dale and Sereno, 1993; Dale, 1999; Fischl and Dale, 2000). Once the cortical models are complete, several deformable procedures can be performed for further data processing and analysis. These procedures include surface inflation (Fischl et al., 1999a)and registration to a spherical atlas that is based on individual cortical folding patterns to match the cortical geometry across subjects (Fischl et al., 1999b). Moreover, parcellation of the cerebral cortex into units with respect to gyral and sulcal structure (Fischl et al., 2004; Desikan et al., 2006) and creation of a variety of surface based data including maps of curvature and sulcal depth are part of the procedure. This method uses both intensity and continuity information from the entire three-dimensional MR volume in segmentation and deformation procedures to produce representations of cortical thickness, calculated as the closest distance from the gray/white boundary to the gray/CSF boundary at each vertex on the tessellated surface (Fischl and Dale, 2000). The maps are created using spatial intensity gradients across tissue classes rather than simply relying on absolute signal intensity. The maps produced are not restricted to the voxel resolution of the original data, and are thus capable of detecting submillimeter differences between groups. Procedures for the measurement of cortical thickness have been validated against histological analysis (Rosas et al., 2002) and manual measurements (Kuperberg et al., 2003; Salat et al., 2004). FreeSurfer morphometric procedures have shown good test-retest reliability across scanner manufacturers and across field strengths (Han et al., 2006; Reuter et al., 2012). A cortical surface-based Desikan-Killiany-Tourville (DKT) atlas (Klein and Tourville, 2012) was mapped to a sphere aligning the cortical folding patterns, which provided accurate matching of the morphologically homologous cortical locations across subjects. For each of the DKT31 protocol-based segments, FreeSurfer calculated 9 different measures, including the number of vertices, surface area, gray matter volume, mean cortical thickness, cortical thickness standard deviation, mean cortical curvature, Gaussian cortical curvature, cortical folding index, and cortical curvature indices (Colby et al., 2012). For the subcortical regions, FreeSurfer calculated the area and volume of the whole segment, white matter volume, and intensity and overall volume of the whole brain divisions, including of the cerebrospinal fluid (CSF), intracranial volume (ICV), gray matter (GM), and white matter (WM). Two of the selected measures are the most common features in structural studies (Arbabshirani et al., 2017). The surface area was calculated by computing the area of every triangle in a standardized spherical surface tessellation. The local curvature was computed using the registration surface based on the folding patterns (Qureshi et al., 2016, 2017b).

#### Cortical and subcortical features

We used measures including the mean cortical thickness, cortical thickness standard deviation, surface area, volume, mean curvature, white matter volume, subcortical segment volume, subcortical intensity, and overall brain volume and intensity as the structural features. All features were acquired as morphological statistics for each subject during the FreeSurfer based preprocessing of the structural MRI data. In addition, after the preprocessing, FreeSurfer's QA Tools were used for the detection and removal of outliers and negative features (Qureshi et al., 2017b).

#### Functional data acquisition

Resting state functional MRI data were collected with single-shot full k-space echo-planar imaging (EPI) with ramp sampling correction using the intercomissural line (AC-PC) as a reference (TR: 2 s, TE: 29 ms, matrix size: 64 × 64, 32 slices, voxel size: 3 × 3 × 4 mm^3^). During the acquisition process, all subjects were instructed to keep their eyes open and stare at the fixation cross.

#### Functional data preprocessing

Preprocessing of functional MRI data was based on Analysis of Functional Neuroimages AFNI software; http://afni.nimh.nih.gov/afni/ (Cox, 1996). Every single EPI volume was coregistered to the corresponding anatomical image of the subject and mapped to the Talairach coordinates space with the TT_N27+tlrc template. We excluded the first six images from each EPI volume to achieve the MR steady state. In addition, slice-timing correction was performed. We censored and excluded time-points based on the number of outliers and head motion magnitude. The same number of slices was excluded for all subjects. Slice alignment was applied using the local Pearson's correlation (LPC) cost function. Correction of head motion along with averaging of EPI volumes was performed to obtain a mean functional image. Each EPI volume underwent linear multiple regression to regress the motion derivatives and effects of the white matter and cerebrospinal fluid. Spatial smoothing was performed using a Gaussian kernel with a blur size of 6-mm full width at half maximum (FWHM). A polynomial detrending was applied (Qureshi et al., 2017b).

#### Global connectivity features

**Figure 1:**
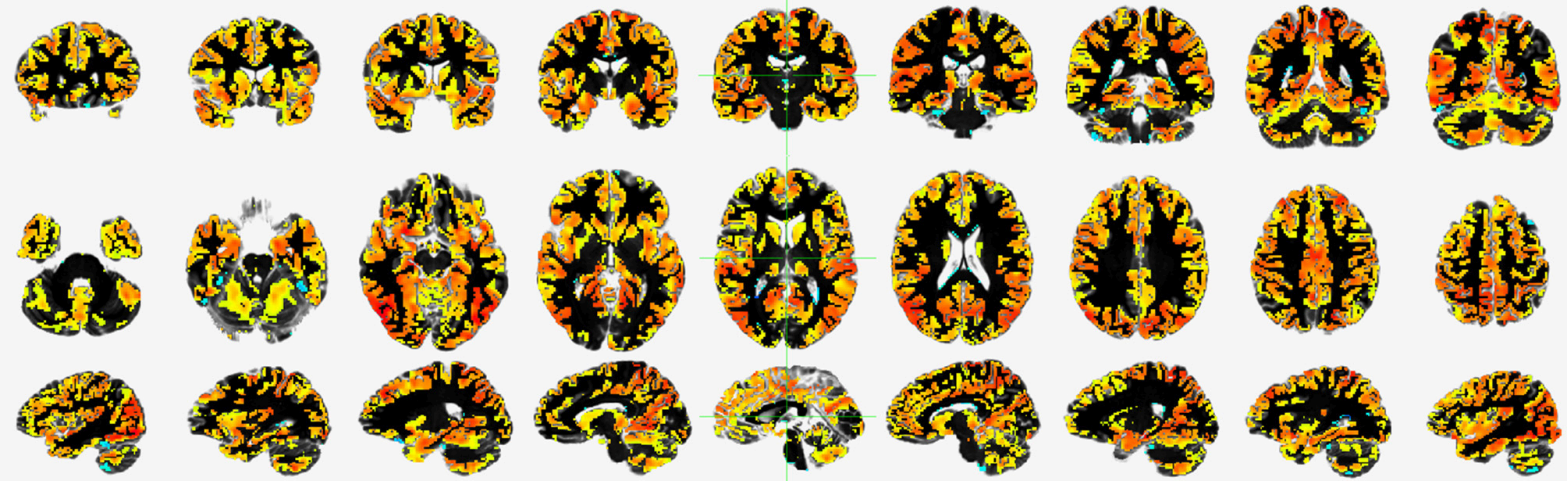
An example of a typical global functional connectivity map for one of the control subject of COBRE dataset. First row depicts the coronal, second row depicts the axial and the third row depicts the sagittal view.

A global connectivity measure was used to calculate the average brain-wise correlation coefficients (GCOR) of all the possible combinations of voxel time series. The GCOR estimation of cortical regions is a computationally expensive process. It involves the calculation of M (M−1)/2 correlation estimates for an M voxel volume (Saad et al., 2013). AFNI simplifies the process by taking reduced time series of each voxel and scaling it by its Euclidean norm. In addition, it averages the scaled time series over the whole brain mask and finally the length *l*_2_ − *norm* of this averaged series represents the GCOR. We used the AFNI program 3dTcorrMap to get the global functional connectivity maps. This involved the calculation of correlation (Pearson's *r*) of the residual time series in each voxel with every other voxel in their brain mask and recording the mean correlation back in the voxel. These connectedness values were further transformed with Fisher's z-transformation to yield normally distributed measures (Gotts et al., 2012). We used the DKD_ Desai_PM Atlas (Desikan et al., 2006) of the AFNI package to acquire the ROI measures from 102 cortical and subcortical regions across the whole brain. Figure 1 depicts a typical global functional connectivity map for one of the control subjects from the COBRE dataset.

#### Group independent component analysis features

**Figure 2:**
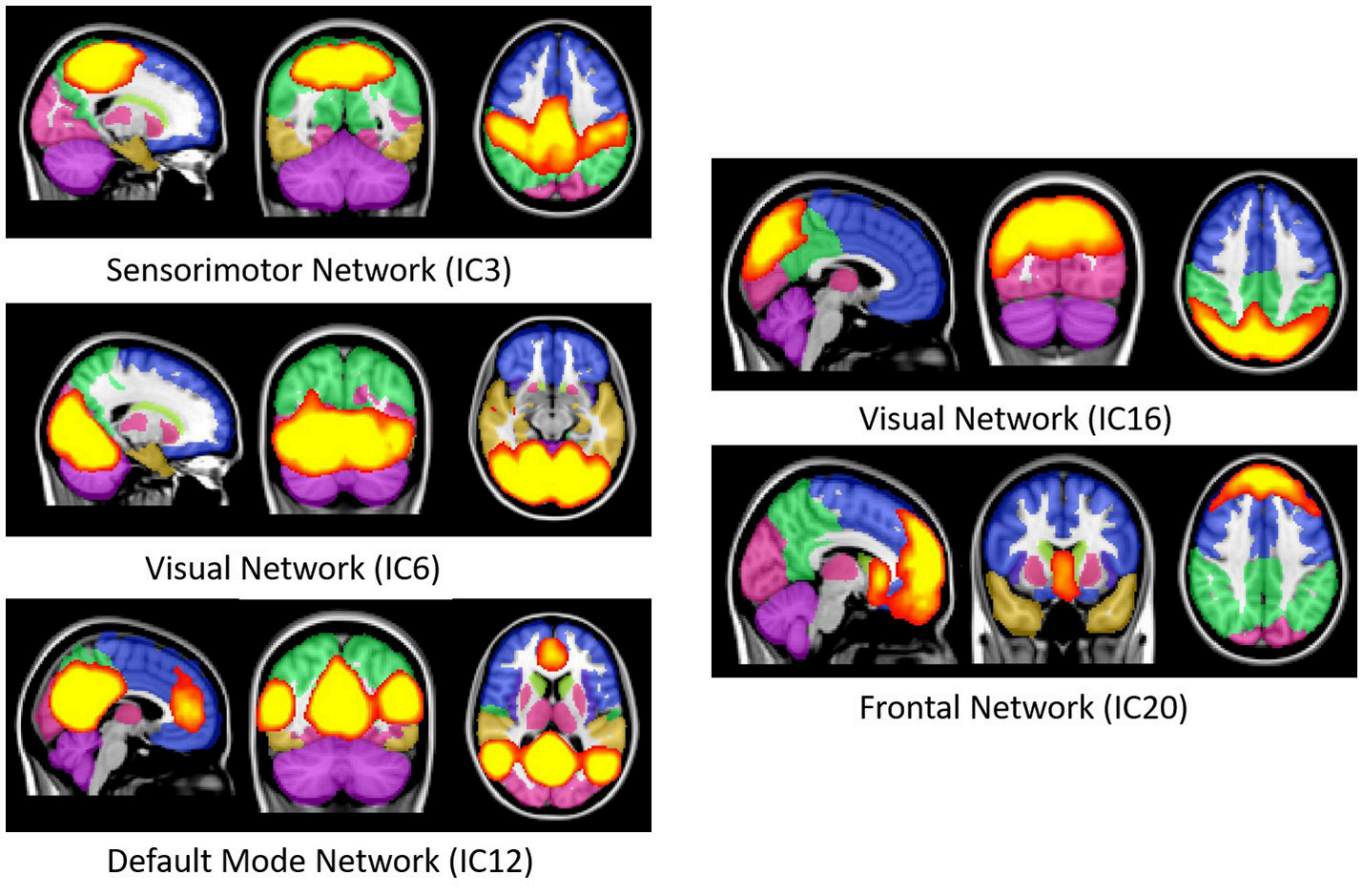
Well-known resting state functional networks acquired from the selected independent component of group ICA result. First image in each network figure depicts the sagittal, second depicts the coronal and third depicts the axial view. The bright yellow color represents the IC's while the pale background colors depicts the MNI152 atlas ROI's. IC, Independent component; ICA, Independent component analysis; MNI, Montreal neuroimaging institute.

Resting state functional MRI data were preprocessed again with FSL (FMRIB Software Library, www.fmrib.ox.ac.uk/fsl) 6.0 for acquiring the group independent component analysis (gICA) based connectivity measures. FSL Multivariate Exploratory Linear Optimized Decomposition into Independent Components (MELODIC) version 3.14 was utilized to perform a single-session ICA. The number of independent components was set as 30, as a model with an order higher than 20 components is usually required for the detection of some components (e.g., S1, S2, striatum), whereas a higher order model shows a decrease in ICA repeatability (Abou-Elseoud et al., 2010). We used variance normalization and thresholded the independent component maps with an alternative hypothesis test that was based on the fitting of a Gaussian/gamma mixture model to the distributions of the voxel intensities within the spatial maps and controlling the local false-discovery rate at *p* < 0.5 (Smith et al., 2004; Beckmann et al., 2005). Among the 30 independent components, 11 were discarded as noise and/or artifacts upon visual inspection by an experienced clinical psychiatrist. Finally, 19 components were identified as functional networks or subnetworks. Similar networks were used in our previous study (Oh et al., 2017). Figure 2 depicts some of the 19 selected components; they represent the well-known resting state functional networks: the default mode network, sensorimotor network, visual network, and frontal network.

**Figure 3:**
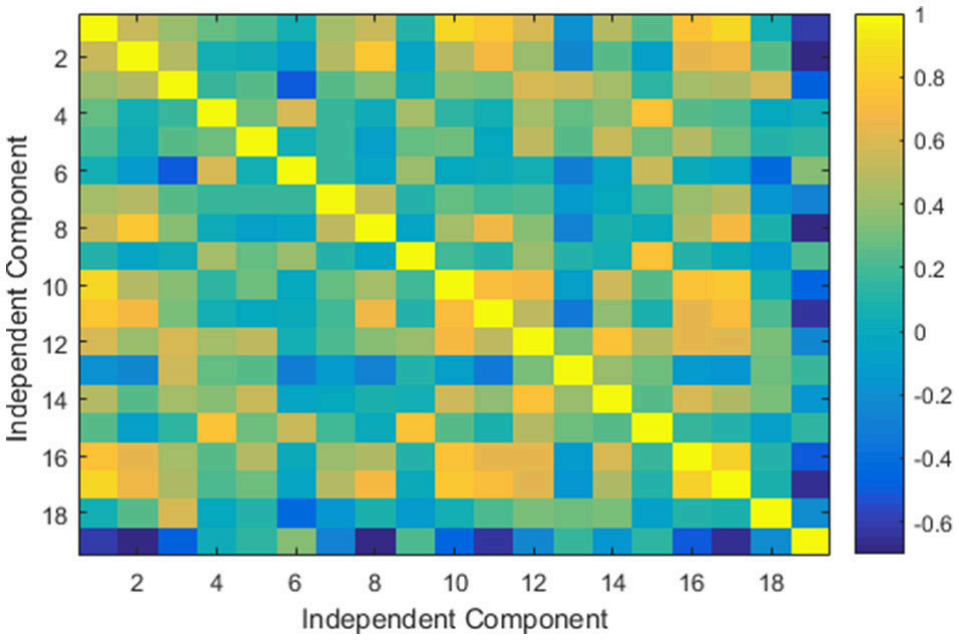
An example of a typical group independent component analysis based connectivity matrix for one of the control subject of COBRE dataset.

Using these components, we applied the FSL dual regression technique (Filippini et al., 2009; Littow et al., 2010; Veer et al., 2010), to acquire subject-specific spatial maps. During this process, we utilized the group spatial maps in a linear model that was fit against the separate fMRI datasets and then acquired the time-course matrices depicting the temporal dynamics for each subject and component. After that, we could acquire the subject-specific spatial maps with the time-course matrices. Next, we calculated the correlation coefficient between the selected 19 components. It resulted in a 19 × 19 correlation based connectivity matrix. We isolated the upper diagonal elements of the connectivity matrix and after vectorizing them, we used them as the gICA features for each subject. Figure 3 depicts a typical ICA connectivity matrix for one of the control subjects from the COBRE dataset.

Finally, we gathered four types of functional connectivity based features, namely: cortical ROI-based global connectivity, subcortical ROI-based global connectivity, whole brain-based global connectivity, and gICA connectivity features. The first three types of features were based on the DKD_ Desai_PM Atlas ROI's (Desikan et al., 2006) and all of them were acquired by considering the average measure of global connectivity of the atlas ROIs.

Table 2 presents a brief overview of the structural and functional measures and number of features used in this study.

**Table 2:** Summary of the features used in this study.

**Label**	**Structural**		**Label**	**Functional**		**Multi-measure and Multi-modal**	
	**Measure**	**Count**		**Measure**	**Count**	**Measure**	**Count**
**CORTICAL FEATURES**							
M1	CT	64	M7	Global connectivity	67	All cortical	449
M2	CT STD	62					
M3	Surface Area	64					
M4	Volume	62					
M5	Mean Curvature	62					
M6	WM Volume	68					
Total	382		67			449	
**SUBCORTICAL FEATURES**							
M8	Volume	26	M10	Global connectivity	24	All subcortical	76
M9	Intensity	26					
Total	52		24			76	
**WHOLE BRAIN FEATURES**							
M11	Volume	27	M10	Global connectivity	11	All whole brain	223
M9	Intensity	14	M12	gICA connectivity	171		
Total	41		182			223	
Grand Total	748						

*CT, Cortical thickness; STD, standard deviation; WM, white matter; gICA, group independent component analysis*.

### Hybrid weighted feature concatenation

Combining features of multiple modalities is a very effective approach for boosting the performance of a machine learning setup. It has been used for machine learning in many research domains, including computer vision for face recognition (Qureshi, 2013), robotics (Naveed, 2014), and in neuroimaging classification (Calhoun and Sui, 2016). We investigated a common feature combination method of simple concatenation in this study, in contrast to the proposed simple and hybrid weighted feature concatenation (WC) method. The proposed method enhanced accuracy while maintaining the number of features similar to that of simple concatenation.

In the proposed method, we calculated the weights of each of 12 (nine structural and three functional) measures *M*_1_, *M*_2_, …, *M*_12_ of the dataset. We want to have the important features to get a high weight *W*_*i*_ such that (0 <= *W*_i_ <= 1), and hence we choose *W*_i_ = 1 − *A*_*max*_ + *A*_i_. Briefly, for calculating the weights *W*_1_, *W*_2_, …, *W*_12_, we first calculated the accuracy of each of the12 measures and then subtracted the individual accuracy *A*_1_, *A*_2_, …, *A*_12_ from the highest accuracy ${\text{A}}_{\text{max}}={\left[{\text{A}}_{\text{max}}\right]}^{\text{T}}$ value among them. Finally, we subtracted the resulting value *R*_1_, *R*_2_, …, *R*_12_ of each measure from vector $\text{1}=\;{\left[1_{1},1_{2},\;..,\;1_{12}\right]}^{\text{T}}$ (that corresponds to perfect classification) to acquire the weights. Mathematically,

$$\begin{array}{l} {\left[R_{1},R_{2},\ldots \;,\;R_{12}\right]}^{T}=A_{max-}\;{{[}_{\;}A_{1},A_{2},\ldots \;,\;A_{12}]}^{T}\\ {\left[W_{1},W_{2},\;\ldots \;,W_{12}\right]}^{T}=1-{\left[R_{1},\;R_{2},\ldots \;,R_{12}\right]}^{T} \end{array}$$

After acquiring the weights [*W*_1_, *W*_2_, … , *W*_12_]^*T*^, in the simple weighted concatenation, the weights of each feature type were multiplied with the corresponding measure data and were concatenated.

$$\begin{array}{l} Simple\;Weighted\;Conatenation=[W_{1}\;*\;M_{1}||\;W_{2}*M_{2}\;||\;,\ldots \;,\;\\ \;||\;W_{12}\;*\;M_{12}] \end{array}$$

Hybrid weighted concatenation was performed by multiplying the corresponding weight with its measure data and dividing the long-concatenated matrix with the sum of the corresponding weights. This step was performed separately for the seven cortical and four subcortical data types regardless of the modality. This division of cortical and subcortical measures was based on the anatomical ROIs acquired from the DKT and DKD_ Desai_PM atlases.

$$\begin{array}{l} \;Cortical\;WC\;=[W_{1}*M_{1}||\;W_{2}*M_{2}\;||\;,...\;,\;||\\ \;W_{7}*M_{7}]\;/\sum_{i=1}^{7}W_{i}\\ Subcortical\;WC\;=[W_{8}\;*\;M_{8}||\;W_{9}\;*M_{9}\;||\;,...\;,\;||\;\\ \;W_{11}*M_{11}]/\sum_{i=8}^{11}W_{i}\\ \;Hybrid\;WC\;=\;[Cortical\;WC||SubcorticalWC||\\ \;gICA\;features] \end{array}$$

Where the symbol “||” corresponds to the concatenation. These simple and hybrid weighted feature concatenation methods were robust and outperformed simple concatenation.

### Classification

Five classifiers were used in this study, namely, ELM, linear and non-linear (radial basis function) SVM, LDA, and random forest ensemble classifiers. In addition, we reported the classification results without applying any feature selection to validate the significance of the proposed hybrid weighted feature concatenation framework. A brief description of all the classifiers used in this study is as follows.

#### Extreme learning machine classifier

The extreme learning machine originally proposed by Huang et al. (2006) has been adopted in many previous neuroimaging studies (Termenon et al., 2013; Zhang and Zhang, 2015; Qureshi et al., 2016), and (Qureshi et al., 2017b) in the binary and multiclass settings. The ELM randomly assigns the weights and bias to the input data to compute the output weight matrix. This random assignment of weights makes the ELM algorithm very fast compared to other gradient-based classifiers such as the SVM. A more detailed discussion of the classifier can be found elsewhere (Huang et al., 2006; Qureshi et al., 2016). The ELM classifier requires the “number of nodes” as the only hyper-parameter that has to be tuned for achieving maximum performance in terms of accuracy. In this study, we used the Matlab implementation of the ELM. A greedy search method was used to tune this parameter for achieving maximum test accuracy. In this study, the search scale for selecting this parameter was set to *N* = [90, 91, …, 220]. A careful selection of the number of nodes is necessary to prevent the classifier from overfitting. All the features were normalized and scaled to values between –1 and 1 for improving the performance of the ELM classifier (Qureshi et al., 2016, 2017b).

#### Support vector machine (SVM) classifier

The SVM classifier, which was originally proposed by Cortes and Vapnik (1995), has been one of the most popular machine-learning tools in the neuroscience domain in the last decade. It is a supervised classification algorithm. It maps features in higher dimensional space using linear and non-linear functions known as kernels. In this study, we used both linear and non-linear (RBF) SVM. In case of SVM-RBF, value of scaling parameter “σ” was tuned in the range σ = [0.000001, 0.00001, …, 100000, 1000000] and box-constraint parameter “C” was tuned in range of *C* = [0.005, 0.01, 0.1, …, 100000, 1000000] using logarithmic grid search method.

#### Linear discriminant analysis (LDA) classifier

The LDA classifier, originally proposed by Duda and Hart (1973), is a linear classifier that utilizes hyperplanes to discriminate the data. These hyperplanes maximize the interclass mean while keeping the interclass variance minimized.

#### Random forest ensemble classifier

Random forest trees, originally proposed by Breiman (1996), are a method of building a forest of uncorrelated trees with randomized node optimization and bagging. Out-of-bag errors are used as an estimate of the generalization error. The random forest (RF) measures variable importance through permutation (Liaw and Wiener, 2002). The general bootstrap aggregation algorithm was used for training. In RF implementation, with decision trees as the learner type, only the number of trees and the number of splits for prediction need to be defined. In this study, we used 30 trees and 20 splits.

### Cross-validation, performance evaluation, and significance testing methods

The classifier performance was measured in terms of nested 10-by-10-fold cross-validated classification accuracy. In the nested cross-validation we repeated each of the 10-fold cross-validation 10 times and reported the average accuracy of the ten 10-fold cross-validation trials. In addition, sensitivity, specificity, negative predictive value, positive predictive value, and F1-Score were calculated as supporting measures for performance evaluation. These measures were calculated through the true positive, true negative, false positive, and false negative values acquired from the confusion matrix by computing the predicted labels with the true labels of the test data. Formally these measures are defined as follows:

$$\begin{array}{l} \;Accuracy\;=\;(TP+TN)/(TP+TN\\ \;+FP+FN)\;\\ \;Sensitivity\;=\;TP/(TP+FN)\\ \;Specificity\;=\;TN/(FP+TN)\\ Negative\;PredictiveValue\;(NPV)\;=\;TN/(FN+TN)\\ \;Positive\;PredictiveValue\;(PPV)\;=\;TP/(TP+FP)\\ \;F1-Score\;=\;2TP/(2TP+FP+FN) \end{array}$$

A Permutation test was used to assess the statistical significance of ELM classifier performance (Golland and Fischl, 2003). Briefly, it works as follows. First, we chose the actual test accuracy as the test statistic of the classifier, the class labels for testing the dataset permuted randomly and were given to the classifier and checked for cross-validation. Generally, the lower the *p*-value of the permuted prediction rate against the prediction rate of the original data labels the higher the significance of the classifier performance. There is no fixed rule for setting the number of permutations. We permuted the data 10,000 times in the current study similar to previous studies (Qureshi et al., 2016, 2017a,b)

**Figure 4:**
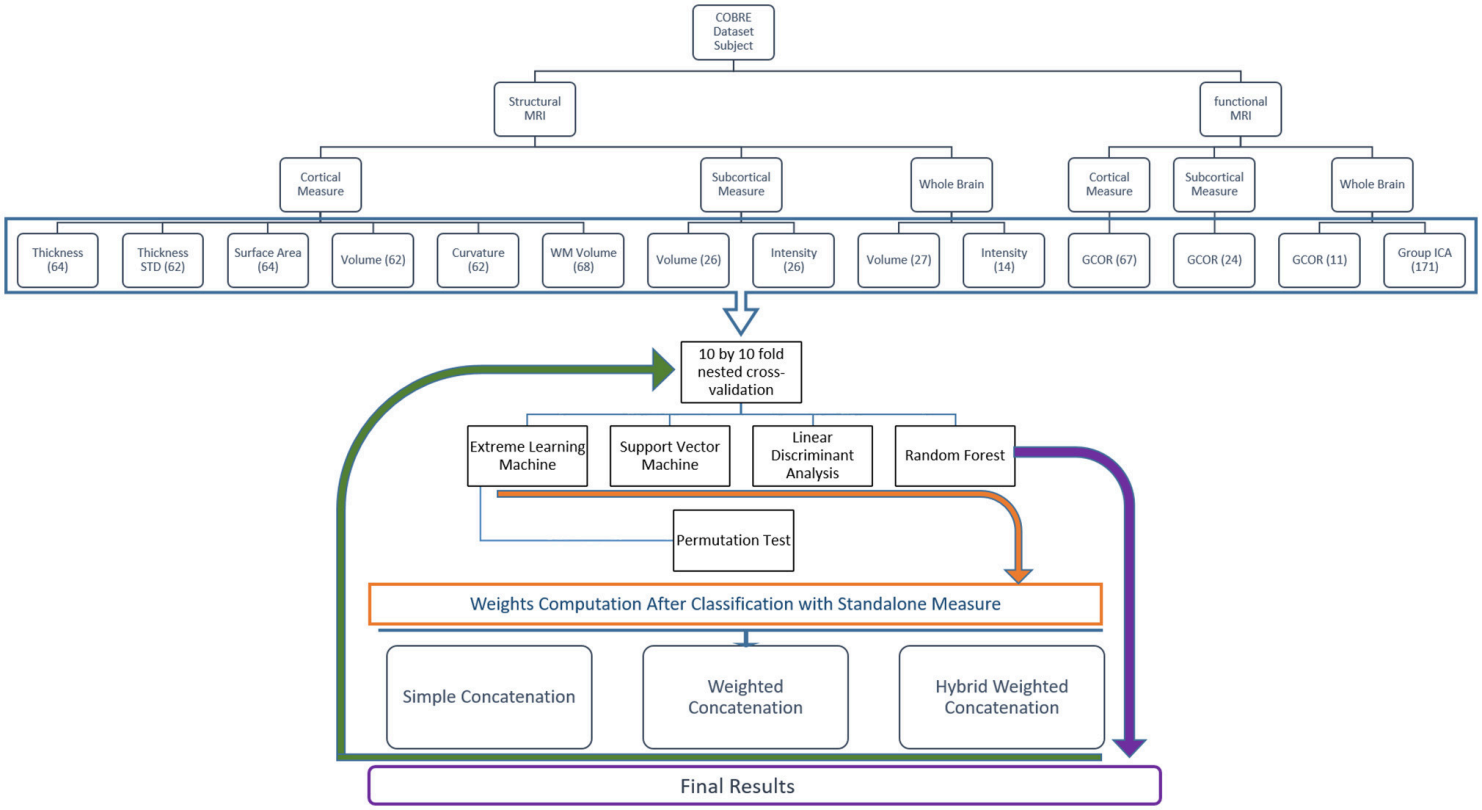
This figure depicts the overall classification feature weighting and concatenation framework.

Figure 4 shows the overall framework of the study including preprocessing, features extraction and hybrid weighted concatenation, and finally classification.

## Results

Results are reported for all the unimodal and multimodal measures for both the simple and hybrid weighted feature concatenation methods in addition to the results of each standalone functional connectivity measure and modality from both the cortical and subcortical regions. The most important result of this study was the highest 10-by-10-fold nested cross-validated predictive accuracy of 99.29% (*p* < 0.0001) by using the hybrid weighted feature concatenation method. In addition, we reported the classification performance of simple concatenation of multimodal cortical and subcortical features. Stand-alone measure accuracies for all the structural and functional features were also reported.

The weight of each measure along with the rank is mentioned in the Table 3.

**Table 3:** Weight and rank of each individual measure.

**Rank**	**Measure**	**Weight**
1	Group ICA	1.0000
2	Curvature	0.9943
3	SC GCOR	0.9829
4	Thickness	0.9819
5	Overall Volume	0.9810
6	Volume	0.9768
7	Cortical GCOR	0.9762
8	Thickness STD	0.9753
9	SC Intensity	0.9695
10	SC Volume	0.9694
11	WM	0.9661
12	Surface Area	0.9518

*ICA, Independent component analysis; SC, subcortical; GCOR, global average functional connectivity; STD, standard deviation; WM, white matter*.

Table 4 summarizes the comparative results of all five ELM, SVM-L, SVM-RBF, LDA, and RF classifiers. Mean testing and training classification scores after 10-by-10-fold nested cross-validation of all the feature groups along with sensitivity, specificity, F1-score, negative predictive value, and positive predictive value measures for all the unimodal and multimodal features are reported for ELM.

**Table 4:** Mean classification performance of multimodal features.

**Classifier**	**Extreme learning machine**								**SVM-L**	**SVM-RBF**	**LDA**	**RF**
**Feature type**	**Train Acc**	**Test Acc**	***p*****-value**	**Sn**	**Sp**	**F1-Score**	**PPV**	**NPV**	**Test Acc**	**Test Acc**	**Test Acc**	**Test Acc**
Hybrid WC	0.9954	0.9929	0.0001	1.000	0.9857	0.9933	1.000	0.9875	0.7780	0.763	0.6810	0.7080
Simple WC	0.9977	0.9804	0.0001	0.9732	0.9875	0.9790	0.9778	0.9875	0.7500	0.750	0.6880	0.7010
Simple concatenation	0.9931	0.9724	0.0001	0.9607	0.9857	0.9723	0.9625	0.9875	0.7710	0.750	0.6880	0.7500
Cortical WC	0.9899	0.9367	0.0001	0.9429	0.9321	0.9359	0.9500	0.9375	0.5960	0.611	0.5830	0.5490
Subcortical WC	0.9931	0.9248	0.0001	0.9571	0.8857	0.9296	0.9639	0.9128	0.6670	0.667	0.6250	0.6040
Cortical concatenation	0.9938	0.9381	0.0001	0.9303	0.9464	0.9367	0.9403	0.9528	0.6320	0.638	0.6110	0.6110
Subcortical concatenation	0.9908	0.9314	0.0001	0.9196	0.9446	0.9309	0.9260	0.9514	0.6390	0.681	0.6180	0.6460
Functional concatenation	0.9961	0.9452	0.0001	0.9304	0.9625	0.9426	0.9375	0.9607	0.6740	0.729	0.6880	0.6740
Structural concatenation	0.9907	0.9319	0.0001	0.9304	0.9357	0.9308	0.9389	0.9417	0.6460	0.625	0.6600	0.6460

*Sn, Sensitivity; Sp, Specificity; SVM-L, linear support vector machine; SVM-RBF, support vector machine with radial basis function kernel, LDA, linear discriminant analysis; WC, Weighted Concatenation; Acc, Accuracy; PPV, positive predictive value; NPV, Negative predictive value; RF, Random Forest*.

**Figure 5:**
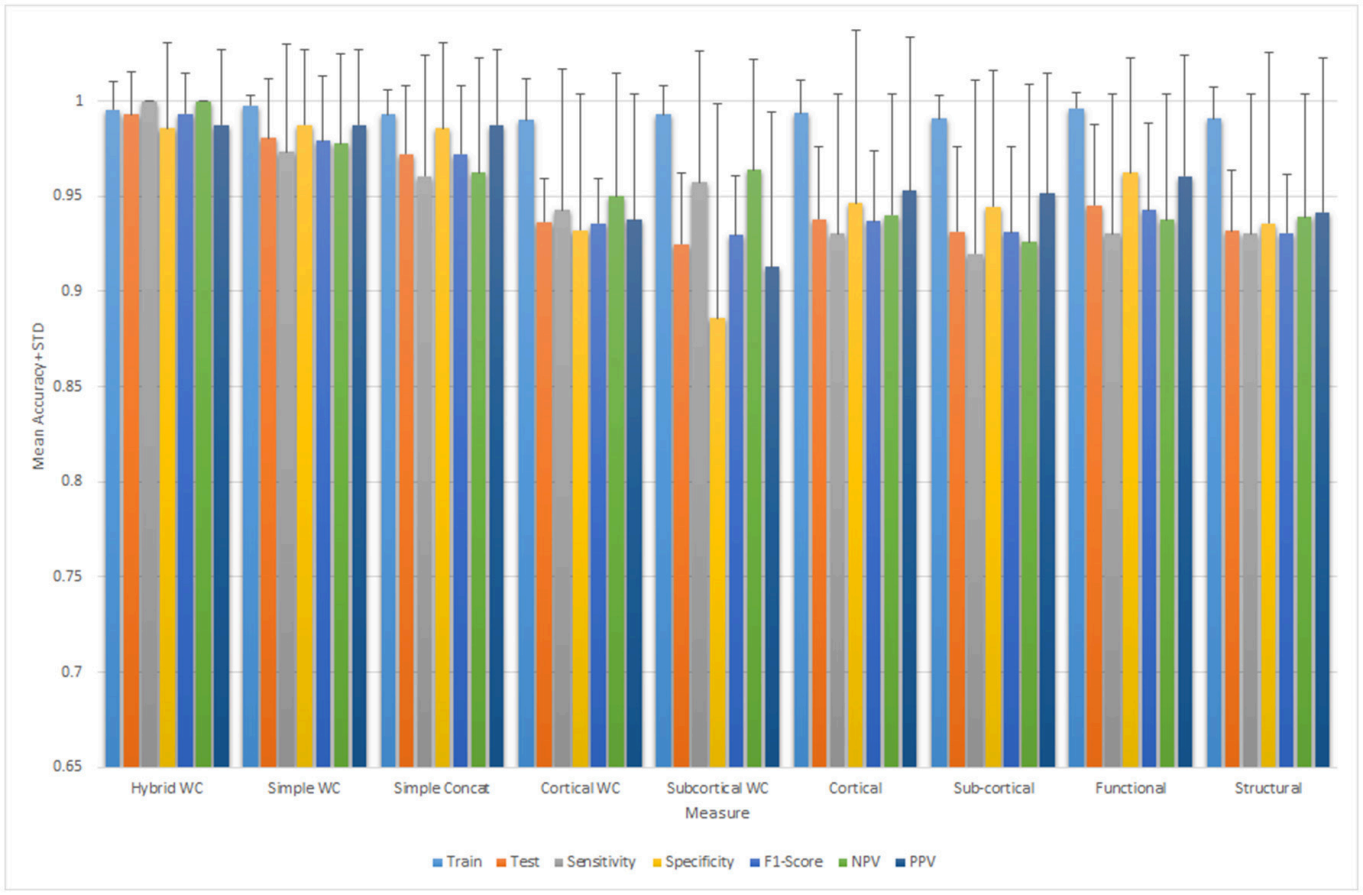
Mean and standard deviation of the classification performance parameters for ELM.

Figure 5 depicts all the mean measures reported in Table 3 along with the standard deviation for the ELM results.

In addition, we calculated the accuracy of all the individual measures for each modality separately. Table 5 summarizes the mean classification scores after 10-by-10-fold nested cross-validation for each individual measure type of structural and functional data.

**Table 5:** Mean classification results of each measure of the data from each modality.

**Classifier**	**Extreme learning machine**								**SVM-L**	**SVM-RBF**	**LDA**	**RF**
**Feature type**	**Train Acc**	**Test Acc**	***p*****-value**	**Sn**	**Sp**	**F1-Score**	**PPV**	**NPV**	**Test Acc**	**Test Acc**	**Test Acc**	**Test Acc**
Thickness	0.9908	0.9114	0.0001	0.9339	0.8929	0.9120	0.9403	0.9024	0.5630	0.598	0.5900	0.6250
Thickness STD	1.000	0.9048	0.0001	0.9000	0.9089	0.8993	0.9175	0.9099	0.6040	0.614	0.5900	0.5070
Surface Area	0.9900	0.8813	0.0001	0.8339	0.9304	0.8736	0.8575	0.9339	0.5420	0.500	0.4720	0.5690
Volume	0.9930	0.9063	0.0001	0.8786	0.9321	0.9010	0.8975	0.9375	0.4580	0.576	0.5140	0.4240
Curvature	0.9961	0.9238	0.0001	0.9339	0.9143	0.9259	0.9403	0.9300	0.5830	0.601	0.5830	0.5560
WM Volume	0.9954	0.8956	0.0001	0.8643	0.9286	0.8916	0.8820	0.9367	0.4720	0.542	0.5070	0.4930
Cortical GCOR	0.9891	0.9057	0.0001	0.9304	0.8839	0.9051	0.9385	0.8913	0.5490	0.591	0.5970	0.6250
SC Volume	0.9745	0.8989	0.0001	0.9018	0.8946	0.8958	0.9157	0.9014	0.7010	0.597	0.6180	0.6250
SC Intensity	0.9930	0.8990	0.0001	0.8589	0.9339	0.8906	0.8864	0.9389	0.6740	0.625	0.6180	0.6320
SC GCOR	0.9884	0.9124	0.0001	0.8750	0.9482	0.9048	0.9008	0.9528	0.5280	0.583	0.5970	0.5760
Overall Volume	0.9861	0.9105	0.0001	0.9161	0.9036	0.9100	0.9228	0.9103	0.6320	0.631	0.5490	0.5630
Group ICA	0.9915	0.9295	0.0001	0.9000	0.9571	0.9269	0.9139	0.9639	0.7150	0.743	0.6940	0.6390

*Sn, Sensitivity; Sp, Specificity; SVM-L, linear support vector machine; SVM-RBF, support vector machine with radial basis function kernel, LDA, linear discriminant analysis; WC, Weighted Concatenation; Acc, Accuracy; PPV, positive predictive value; NPV, Negative predictive value; GCOR, global average functional connectivity; ICA, independent component analysis; WM, white matter; STD, standard deviation; SC, subcortical; RF, random forest*.

**Figure 6:**
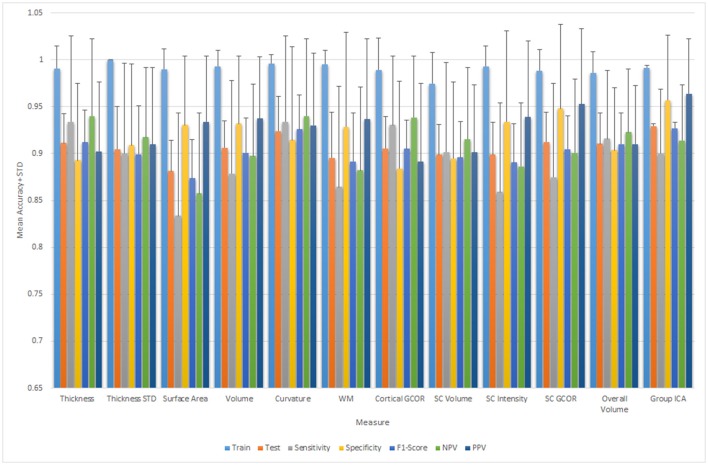
Mean and standard deviation of the classification performance parameters for ELM.

Figure 6 depicts all the mean measures reported in Table 4 along with the standard deviation for the ELM results.

## Discussion

This study reports the multimodal hybrid weighted feature concatenation of neuroimaging data for the purpose of classification. We proposed a simple and powerful hybrid weighted feature concatenation method. Two types of functional connectivity based features were utilized in this study to acquire the highest classification accuracy. Group independent component analysis (gICA) of functional connectivity features is a very robust stand-alone measure for achieving high classification accuracy and provides an accuracy as high as 92.95% (*p* < 0.0001) when used with an extreme learning machine. In addition, our highest prediction result of 99.29% (*p* < 0.0001) accuracy shows that the proposed hybrid weighted features concatenation method is very effective for boosting the accuracy of the ELM classifier.

The ELM classifier in combination with the proposed hybrid weighted feature concatenation method is effective for the classification of neuroimaging data. A few recent studies such as Qureshi et al. (2016), Qureshi et al. (2017a), and Qureshi et al. (2017b) were based on an extreme learning machine classification framework for neuroimaging data. However, the current study proposed a very simplified hybrid weighted feature concatenation method that allows for the acquisition of higher classification accuracies without requiring any kind of feature selection approach. To wit, this advanced concatenation method is time-effective while boosting classification performance.

### Comparison to previous studies

For the purpose of comparing the efficiency of the method proposed in the current study, we enlisted all previous studies on multimodal classification of Schizophrenia in Table 6, to the best of our knowledge.

**Table 6:** Comparison with the classification scores of previous studies using multimodal data.

**Study reference**	**Modality**	**Sample size**	**Features**	**Feature count**	**Classifier**	**Accuracy (%)**
Proposed Method	sMRI + fMRI	144	Structural ROI measures, Global functional connectivity, Group ICA	748	ELM	99.29
Du et al., 2012	rs-fMRI + task-related fMRI (auditory oddball task)	56	Kernel PCA with spatial ICA maps	53	Fisher's linear discriminant	98
Silva et al., 2014	sMRI + fMRI	144	Gray matter density based ICA features (sMRI), group ICA features (fMRI)	410	Gaussian process classifier	94
Cetin et al., 2016	rs-fMRI + task-related fMRI (auditory oddball task) + MEG	55	Group ICA based functional connectivity scores, MEG data for each frequency	103	LDA, Naïve Bayes classifier, and non-linear SVM	90
Ota et al., 2013	sMRI + diffusion MRI	50	Volume and fractional anisotropy (FA) of certain regions (insula, thalamus, ACC, ventricles, and corpus callosum)	31	Linear discriminant analysis	88
Yang et al., 2010	task-related fMRI (auditory oddball task) + SNP (genetic data)	40	Voxels in the fMRI map, ICA components, SNPs	411	Support vector machine	87
Sui et al., 2013	sMRI + rs-fMRI + diffusion MRI	63	GM density, ALFF (amplitude of low-frequency fluctuation), FA	1,863	Support vector machine	79

To date, numerous classification studies have been conducted using only one neuroimaging modality to discriminate patients with schizophrenia from healthy controls (Fan et al., 2005, 2008; Bassett et al., 2012; Zanetti et al., 2013). As shown in Table 6, we listed all previous classification studies that used schizophrenia vs. normal control multimodal data, and some of these studies achieved high classification accuracy. However, a relatively small sample size can boost accuracy by chance, therefore, more than 130 subjects were reportedly required to perform such a study (Nieuwenhuis et al., 2012). In fact, only one of these studies fulfilled this criterion (Silva et al., 2014). The result of this study was good (94% accuracy); however, the number of included feature types was smaller than that of the present study. For example, we utilized global and group ICA-based connectivity features from the fMRI data as well as other structural measures including surface area, curvature, and white matter information. Considering that additional features, such as surface area, can be related to disease progress, the use of more features might have contributed to the higher accuracy of our classification (Li et al., 2016). White matter information, in particular, should not be ignored as there is evidence that schizophrenia is a disorder involving both gray and white matter abnormalities (Davis et al., 2003; Kubicki et al., 2005).

### Clinical significance of the results

To summarize, our results showed that the highest level of classification accuracy may be obtained when integrating all structural information including gray and white matter, with functional connectivity information such as ICA and global connectivity. It may mean that, several parts of the brain experience deterioration, both structurally and functionally, as a result of schizophrenia, thus, classification using machine learning should include the maximum amount of information computational capacity permits. The DSM criteria, by which schizophrenia is diagnosed, is still not perfect. The population of patients with schizophrenia is heterogeneous, as the diagnosis is based on observable symptoms, and that presents a challenge for diagnosing or representing all implicated characteristics by a single type of feature. In fact, since we did not adopt a feature selection process during the analysis, it is not known which features influenced the classification. A gross estimation may be performed by observing feature measure weight information as provided in Table 3, where group ICA features appear as the most significant contributor to the classification, although all other features also exhibited ~90% accuracy. These results may support the well-known findings that schizophrenia is associated with comprehensive abnormalities in both structural and functional domains. However, in terms of ranking importance, functional connectivity information showed slightly superior performance than structural information, and with regard to structural information, cortical information was superior to subcortical information in classification performance. Taken together, it appears that aberrant functional connectivity and structural abnormalities in the cortex should be given more weight, which is in line with recent research findings (Dong et al., 2017; Larivière et al., 2017; Massey et al., 2017; Mørch-Johnsen et al., 2017; Peters et al., 2017).

In real clinical practice, the misdiagnosis related to schizophrenia usually happens due to many other psychiatric diseases which can show the similar psychotic symptoms such as mood disorders and personality disorders. In addition, the person in the premorbid or prodromal stage of psychosis who can almost fulfill the diagnostic criteria of schizophrenia confuses the clinicians about whether they should be diagnosed with schizophrenia or not. In this study, the high-risk groups or groups with other psychiatric diseases were not included, therefore our classifier has some limitations to be used directly in clinical settings because it can only differentiate healthy people and schizophrenia patients. Therefore, other classifiers (bipolar disorder vs. healthy control, bipolar disorder vs. schizophrenia, or multiclass classifier) should be developed and ameliorated to give more information to the clinicians. Considering the machine learning based diagnosis is not perfect yet, it should be used only as a supportive tool in conjunction with a standard diagnosis. However, it can be really helpful when the diagnosis is difficult.

## Limitations

First, our classification process underwent nested cross-validation without novel data for testing. Although we acknowledge this issue, we sought to use as much training data as possible to overcome the sample size limitations in the classification. Second, the symptom severity score (PANSS) showed that our sample's symptoms were relatively mild, however, the patients had already experienced active symptoms, therefore, it was challenging to use our classifier for purpose of prediction. If we had included a high-risk group, we could have utilized our classifier for early prediction.

In addition, it appears it is relatively easier to distinguish patients from healthy controls than to distinguish patients from high-risk groups. Considering our classifier was only learned to distinguish non-healthy brains from healthy brains, it has limited role in real clinical field. In other words, other classifiers using the data from other groups, such as bipolar disorder and high-risk groups for schizophrenia, would be more helpful. Those classifiers would enable the identification of schizophrenia-specific features and could perform the differential diagnosis among many disorders that involve structural or functional brain abnormalities. However, despite of those limitations, we believe that high accuracy of our classifier would be useful in some limited clinical situations.

## Conclusion

The proposed multimodal hybrid weighted feature concatenation method is robust, simple, and straightforward, and provides promising results. The extreme learning machine is an excellent choice for classifying neuroimaging data. Multimodal and multi-measure features are robust for providing higher accuracy. The group ICA based functional connectivity features method is appropriate and exhibits the highest discriminatory power as a stand-alone measure type. We did not utilize any feature selection and optimization algorithm in this study, but as the chosen features had extremely highly discriminatory power, the present study produced clinically acceptable diagnostic accuracies.

## Author contributions

MQ and JO have contributed equally to this work. MQ and JO selected the subjects for balanced experiment design from the publicly available COBRE database. MQ pre-processed the data, developed, and applied the proposed feature concatenation methods and classified the data. JO have drafted the introduction and discussion sections. DC, HJ, and BL helped in drafting the manuscript with MQ and JO. BL supervised the entire research process and revised the manuscript for publication. All authors contributed to the research design, results interpretation, and proofreading of the final manuscript.

### Conflict of interest statement

The authors declare that the research was conducted in the absence of any commercial or financial relationships that could be construed as a potential conflict of interest.
